# Combined optogenetics and voltage sensitive dye imaging at single cell resolution

**DOI:** 10.3389/fncel.2014.00311

**Published:** 2014-10-08

**Authors:** Silvia Willadt, Marco Canepari, Ping Yan, Leslie M. Loew, Kaspar E. Vogt

**Affiliations:** ^1^Neurobiology/Pharmacology, Biozentrum, University of BaselBasel, Switzerland; ^2^Laboratoire Interdisciplinare de Physique (CNRS UMR 5588) and Grenoble Institut des Neurosciences (Inserm U836)Grenoble, France; ^3^R. D. Berlin Center for Cell Analysis and Modeling, University of Connecticut Health CenterFarmington, CT, USA; ^4^International Institute for Integrative Sleep Medicine (IIIS), University of TsukubaTsukuba, Japan

**Keywords:** optogenetic stimulation, voltage sensitive dye imaging, dendritic processing, synaptic integration, GABAergic transmission

## Abstract

Information processing in the central nervous system makes use of densely woven networks of neurons with complex dendritic and axonal arborizations. Studying signaling in such a network requires precise control over the activity of specific neurons and an understanding how the synaptic signals are integrated. We established a system using a recently published red-shifted voltage sensitive dye in slices from mice expressing channelrhodopsin (Ch) in GABAergic neurons. Using a focused 473 nm laser for Ch activation and 635 nm laser wide field illumination for voltage sensitive dye excitation we were able to simultaneously measure dendritic voltage transients and stimulate inhibitory synaptic connections. The combination of these techniques provides excellent spatiotemporal control over neuron activation and high resolution information on dendritic signal processing.

## Introduction

Information processing in neural networks chiefly relies on the interplay of excitatory and inhibitory synaptic transmission between nerve cells. These synaptic signals are then integrated in the complex dendritic trees of the neurons in the network and determine their output patterns. The study of a given neural network therefore should encompass the controlled activation of specific neurons and a way to measure how synaptic signals affect target neurons. Classical methods, using electrical stimulation and somatic whole cell recordings provide important insights into the organization of a neuronal network, but often fail to reveal its deeper complexity. First, dendritic processing is not directly visible at somatic recording sites. Second, controlled activation of identified neurons requires paired recordings, which rely on intact connections between patched somata and their axons, thus severely limiting the investigation of long-range connections.

**Figure 1:**
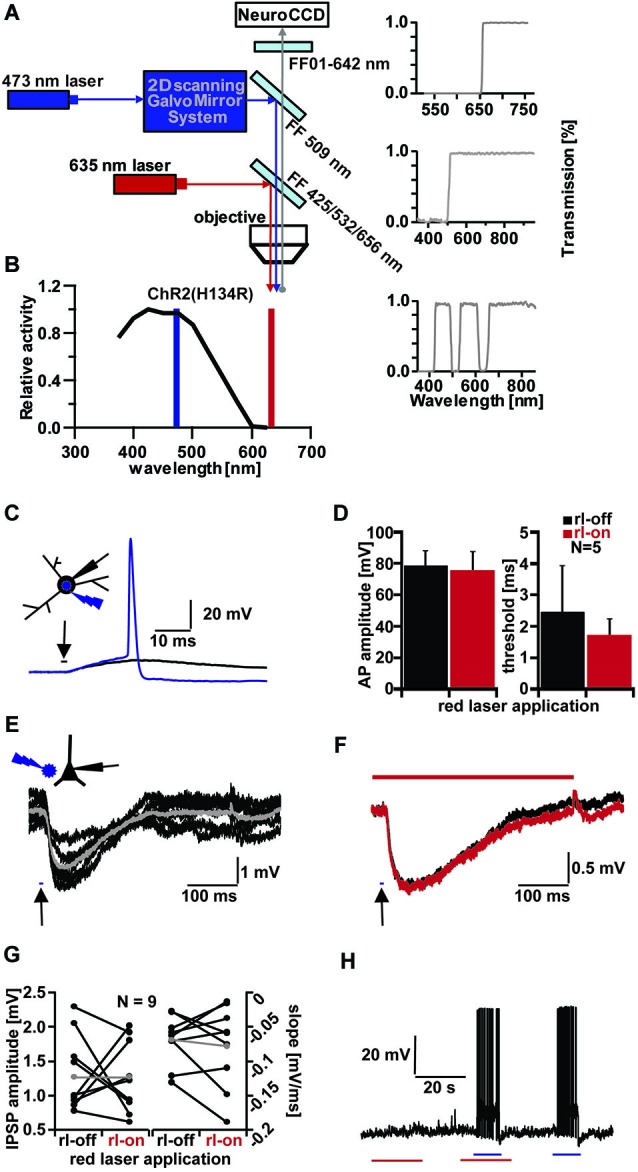
**Channelrhodopsin (ChR) activation and influence of red laser (rl) application. (A)** Schematic drawing of optical system for combined VSD imaging and ChR2 activity modulation. **(B)** ChR2 light excitation spectra (adapted from Gradinaru et al., 2010). Blue line indicates wavelength of 473 nm-laser application, red line indicates 635 nm-laser application. **(C)** Testing threshold of ChR2+ interneurons activation by increasing light duration (in 0.1 ms increments) applied to soma with below threshold (black trace, 2.5 ms) and above threshold duration (blue trace, 2.6 ms; Vm = −55 mV); inset shows light application configuration on patched Stratum radiatum interneuron. **(D)** Influence of rl intensity used for VSD imaging on AP amplitude and light duration threshold of five ChR2+ interneurons. Average amplitude (left bar graph): 78.41 ± 9.77 mV (rl-off, black), 75.2 ± 12.21 mV (rl-on, red). Average threshold (right bar graph): 2.46 ± 1.46 ms (rl-off, black), 1.68 ± 0.56 ms (rl-on, red). **(E)** Evoked IPSPs in a CA1 pyramidal cell by 10 ms blue light application (arrow) next to cell in layer pyramidale (schematic inset). Averaged IPSP (gray; ΔIPSP = 2 mV) of 6 traces (black; Vm = −60 mV). **(F)** Comparison of evoked IPSPs without (black trace, average of 8) and with rl (red trace, average of 9) application (Vm = −61 mV, ΔIPSP = 1.2 mV). **(G)** Influence of rl application on IPSP amplitude (left bar graph) and slope (right bar graph) of CA1 pyramidal cells. Average IPSP amplitude (gray): 1.27 ± 0.5 mV (rl-off, left), 2.3 ± 1.99 mV (rl-on, right). Average slope (gray): −0.07 ± 0.04 mV/ms (rl-off, left), −0.18 ± 0.2 mV/ms (rl-on, right). **(H)** Influence on membrane potential (Vm = −62 mV) of ChR2+ interneuron with red (red line, 20 ms) and blue laser (blue line, 10 ms) application.

The novel method of optogenetics, the expression of light-sensitive proteins such as channelrhodopsin (ChR) in select subtypes of neurons and their precisely controlled activation in time and space by light have become important for understanding the function of particular neurons (Boyden et al., 2005; Zhang et al., 2007). Long-range connections can be investigated by selectively stimulating ChR expressing axons (Deisseroth, 2011). Single cell voltage-sensitive dye (VSD) imaging (Zecević, 1996) has the potential to reveal dendritic signal integration of both excitatory (Canepari and Vogt, 2008) and inhibitory (Canepari et al., 2010) synaptic potentials at high resolution. Due to the broad absorption spectrum of ChRs (Figure 1B) the combination of optogenetics and other imaging techniques such as VSD imaging remain challenging. The most commonly used dyes, have to be excited at high intensity at wavelength which will overlap with the activation spectrum of ChRs. Recently, a new class of VSDs with red-shifted absorption spectra have become available (Wuskell et al., 2006; Kee et al., 2008; Yan et al., 2012). Successful experiments using bulk loading of these novel dyes combined with optogenetics have been reported (Leão et al., 2012; Tsuda et al., 2013). Here we show the results of successfully combining high resolution VSD imaging using patch-pipette loading of the recently published dye Di-2-ANBDQPTEA and optogenetics in acute brain slices.

## Materials and methods

### Brain slice preparation

All experiments were approved by Basel cantonal veterinary authorities. Recordings were performed in 300 µm thick brain slices from juvenile (22–32 day old) VGAT-ChR2-EYFP mice that expressed functional ChR2(H134R) under the control of vesicular γ-aminobutyric acid (GABA) transporter (VGAT) promoter elements specifically in GABAergic neurons in the nervous system (Zhao et al., 2011). After deep isoflurane anesthesia mice were decapitated and transversal hippocampal slices were cut using a vibrating microtome (VT1200S, Leica, Switzerland). Slicing was performed in ice-cold solution containing (in mM) NaCl 87, Sucrose 75, Glucose 25, NaHCO_3_ 25, MgCl_2_ 7, KCl 2.5, NaH_2_PO_4_ 1.25, CaCl_2_ 0.5, equilibrated with 95% O_2_ and 5% CO_2_. After cutting, slices were incubated at room temperature for 30 min in artificial cerebrospinal fluid (ACSF), also used as extracellular solution for the experiments. This solution contained (in mM): NaCl 125, NaHCO_3_ 26, NaH_2_PO_4_*H_2_O 1.25, KCl 2.5, MgSO_4_ 1.0, CaCl 2.5. The osmolarity was adjusted to 300–310 mOsmol and the pH was maintained at ~7.4 when bubbled with a gas mixture containing of 95% O_2_ and 5% CO_2_.

### Neuronal loading and electrophysiology

For imaging experiments, acute brain slices were transferred into a holding chamber perfused with a constant flow of ACSF at about 1 ml/min at room temperature.

CA1 pyramidal cells or stratum radiatum interneurons were loaded by whole-cell patch recordings with the VSD Di-2-ANBDQPTEA (PY3283; available from L.M. Loew; Yan et al., 2012) diluted in the intracellular solution. Dye concentration was ~1–2 mg/ml, and cells were held in the whole cell configuration for ~40 min to allow for dye diffusion. Other loading parameters were as described in more detail previously (Canepari et al., 2008, 2010).

The KMeSO_4_-based intracellular solution contained (in mM): 5 Na-ATP, 0.3 Tris-GTP, 14 Tris-phosphocreatine, 20 HEPES, 125 KMeSO_4_ and 5 KCl; 285 mOsmol and pH 7.35 adjusted by KOH titration. We used borosilicate electrodes for whole-cell patch-clamp recordings (1.5 mm external diameter, 1.17 mm internal diameter) without filament and an open tip resistance of 5–6 MΩ. Background fluorescence increases due to dye spillage was avoided by tip-filling the electrode with dye-free solution. In addition, before reaching cells, positive pressure in the pipettes was kept low and controlled with a manometer at ~5 mbar (Model 840081; Sper Scientific, Scottsdale, AZ).

While staining cells with VSD, in some experiments somatic whole-cell recordings were performed using a Multiclamp 700 A amplifier (Axon Instruments, Germany) and an upright microscope (Olympus BX51-WI, Olympus, Switzerland).

Staining time (~40 min), was determined by measuring the resting fluorescence from the cell body at reduced excitation light intensity. After sufficient dye had diffused into the cell, pipettes were gently removed by forming outside-out patches. Optical and whole-cell re-patch recordings were performed when dendrites were sufficiently filled with VSD (~30–40 min after loading pipette removal).

Extracellular stimulation was performed by using borosilicate patch pipettes filled with ACSF. Feedforward inhibition was evoked by stimulation of Schaffer collaterals in stratum radiatum at the border between CA3 and CA1.

Stimulation pulses were of 0.1 ms duration and their intensity varied between 20 to 80 µA. Pulses were delivered by an IS4 stimulator (SC-Devices, Switzerland) and triggered by stimulation protocols written in IGOR Pro software (Wave Metrics, USA).

Somatic electrode recordings were acquired at 16 kHz and filtered at 4 kHz by using the Redshirt imaging system or acquired at 20 kHz and filtered at 2 kHz by a separate A/D board (NI USB-6343, National Instruments, Switzerland).

### Optical recordings and light ChR2-Stimulation

The VSD Di-2-ANBDQPTEA was excited by using a 635 nm–500 mW solid-state laser (MLL-III-635, Changchun New Industries, P.R. China) at 50% intensity to excite the dye at the border of its absorption spectrum (100 nm red shifted from its absorption peak), increasing its dynamic range (Kuhn et al., 2004). Wide field illumination was achieved by focusing the light onto the back focal plane of the microscope objective using a commercially available single port epifluorescence coupler (Till Photonics, Gräfelfing, Germany), light intensity at that spot was 100 mW. Homogeneity of illumination was tested and optimized by imaging a uniformly fluorescent target.

Optical signals were captured with a high-speed, 80 × 80 pixel CCD camera (NeuroCCD-SM, RedShirtImaging LLC, China) at frame rates of 500 Hz. The fluorescence image of the cell was projected via a 0.2 fold optical coupler onto the CCD camera. Camera gain was set to 10-fold. The imaged field in our measurements was ~125 µm × 125 µm. Fractional changes in optical signal intensities (DF/F) were analyzed from either several regions of interest (ROIs) along the stained dendrite by averaging 20–60 pixels or averages over the whole visible dendrite (average pixel ∑ = 180), at a pixel size of 1.56 µm × 1.56 µm. To improve the signal-to-noise ratio, depending on the type of experiment averages of 2–10 trials were taken (the numbers are indicated in the respective figure legend).

ChR2 activation was achieved by blue laser light (473 nm) stimulation of labeled interneurons. The light spot (~30 µm diameter) of a focal illumination system was positioned on the slice surface via a 2D scanning galvo-mirror-system controlled by protocols written in IGOR Pro software (Wave Metrics, USA). Square pulses of light between 2–10 ms, depending on type of experiment and cell were used.

To separate the light paths of the two excitation wavelengths and red emission light we constructed a stacked filter set. 635 nm VSD excitation was directed towards the slice using a triple edge dichroic beamsplitter (FF425/532/656-Di01, Semrock Inc., USA), which at the same time permitted passage of 473 nm blue excitation wavelength (Figure 1A). The 473 nm ChR2 excitation light was directed towards the preparation using a single edge dichroic beamsplitter (FF509-Di01, Semrock Inc., USA). VSD emission light was finally filtered with a 642 nm long pass filter (FF01-642, Semrock Inc., USA).

Slices were visualized and imaged using a 60x high aperture water immersion lens (Olympus 60x/1.1 NA, Olympus, Switzerland).

### Analysis

Optical signals were analyzed as fractional changes of fluorescence (DF/F). Optical and electrophysiological recordings were analyzed with dedicated software written in MATLAB (The MathWorks). Optical signals were corrected for the bleach fraction, by fitting a monoexponential function to data from trials without electrical or optical stimulation and subtracting this function from trials with stimulation. Optical and electrical stimulation artifacts were truncated for clarity purposes.

Statistics were calculated in Excel (Microsoft Office 2010) and averages are presented as the mean ± standard error of the mean (SEM). Statistical significance was assessed using paired Student’s *T*-test and reported as *p*-values, unless indicated otherwise.

## Results

The two excitation wavelengths (blue 473 nm for ChR2 stimulation and red 635 nm for dye excitation) were combined using an optical system as shown (Figure 1A), while a single emission wavelength was projected onto the chip of a high-speed CCD camera. Focused illumination with a spot diameter of ~30 µm in the focal plane of the preparation at 473 nm was steered with a pair of galvanometer-controlled mirrors, which allowed the beam to be freely positioned in the field of view. Bright field illumination at 635 nm was coupled into the beam path with the help of a triple band dichroic mirror, which permitted the 473 nm light to pass.

We first tested the ability of the focused blue laser illumination to activate interneurons expressing ChR2(H134R) under the control of the vesicular GABA transporter (VGAT; Zhao et al., 2011). Brief pulses of blue light reliably produced depolarizing responses in interneurons (Figure 1C). Increasing the duration of the pulses evoked action potentials in all interneurons tested (*N* = 7).

To successfully combine any imaging technique with ChR2 stimulation, the wavelength and intensities of the excitation light have to be chosen such as to avoid spurious ChR2 activation by imaging. We therefore tested, whether illuminating the preparation with 635 nm red-light at the intensity used during imaging, affected the behavior of ChR2(H134R) expressing cells. We compared the size and threshold of 473 nm-evoked action potentials with and without red laser (rl) light (Figures 1D,H). Action potential amplitudes showed no significant differences between the two conditions (rl-off: 78.41 ± 9.77 mV; rl-on: 75.2 ± 12.21 mV; *p* = 0.66, *N* = 5). In addition, the time of light application to reach the threshold for action potential-induction (rl-off: 2.46 ± 1.46 ms; rl-on: 1.68 ± 0.56 ms; *p* = 0.31, *N* = 5) was not different with or without red light illumination. To illustrate that the ChR2(H134R) cells were inert to red light, we recorded their membrane potential for extended periods with intermittent red illumination. We applied alternately 635 nm light for 20 s alone, subsequently combined with 10 ms 473 nm light and finally only 473 nm light for 10 ms (Figure 1H). There was no significant change in the membrane potential in these cells due to red light illumination (−0.17 ± 1.0 mV, *N* = 5) and all the cells fired reliably to 473 nm flashes both under control condition and concurrent red light illumination. These results indicate that 635 nm illumination neither directly activated ChR2, nor interfered with ChR2 activation by 473 nm laser pulses.

Short-latency inhibitory postsynaptic potentials (IPSPs) were observed in hippocampal pyramidal cells following blue light stimuli directed either at interneurons in their vicinity or at the surrounding neuropil (Figure 1E), indicating that GABA release could be triggered both by somatically and axonally expressed ChR2. On average, blue light pulses of 11.7 ± 5.6 ms (*N* = 9) duration had to be applied to induce detectable IPSPs in principal cells.

Analogous to the light-induced action potentials of interneurons, the light-evoked IPSPs in pyramidal cells were not significantly changed by rl light exposure (Figures 1F,G). Representative blue light-evoked IPSPs are shown (Figure 1F) without (black, rl-off) and with rl light (red, rl-on). IPSP amplitudes were 1.33 ± 0.55 mV in control and 1.27 ± 0.53 mV under red illumination (*p* = 0.82, *N* = 9), while their slopes were −0.07 ± 0.04 mV/ms in control and −0.08 ± 0.06 mV/ms under red illumination (*p* = 0.56, *N* = 9).

**Figure 2:**
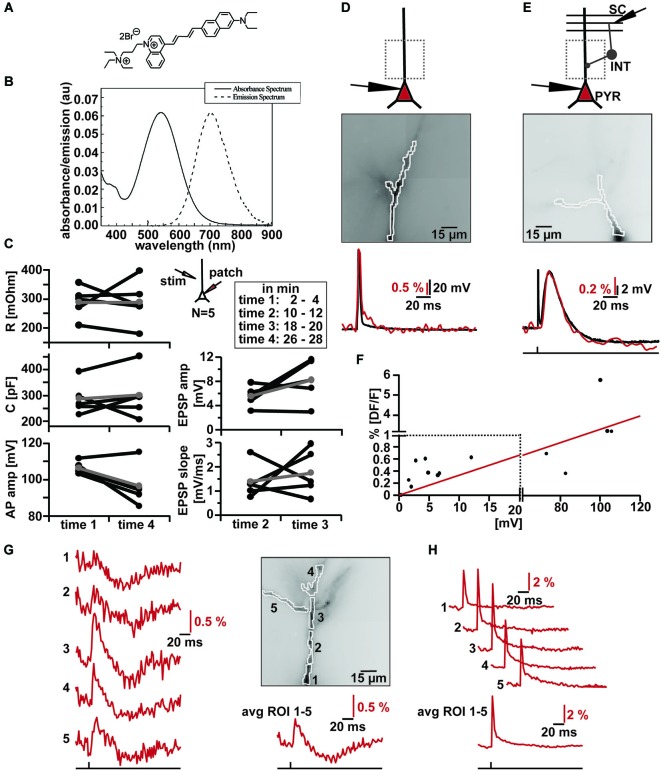
**VSD imaging using the new blue-dye DI-2-ANBDQPTEA. (A)** Molecular structure of DI-2-ANBDQPTEA (Yan et al., 2012). **(B)** Absorbance and emission spectra of DI-2-ANBDQPTEA. **(C)** Changes in cell characteristics during dye loading by whole-cell patch clamp in current clamp (~Vm = −60 mV, *N* = 5) and extracellular stimulation (inset illustration). Measured at time 1 (2–4 min) and time 4 (26–28 min), as well in between time 2 (10–12 min) and time 3 (18–20 min). Average input resistance (red; left, top panel): 289.33 ± 54.09 MΩ (time 1), 288.83 ± 78.33 MΩ (time 4) *p* = 0.99; average cell capacitance (red; left middle panel): 287.75 ± 63.41 pF (time 1), 300.48 ± 91.53 pF (time 4), *p* = 0.68; average action potential amplitude (red; left, bottom panel): 106.37 ± 3.47 mV (time 1), 96.47 ± 11.18 mV (time 4), *p* = 0.07; average EPSP amplitude (red; right top panel): 5.51 ± 1.71 mV (time 2), 8.18 ± 3.55 mV (time 3), *p* = 0.15; average EPSP slope (red; right bottom panel) 1.39 ± 0.71 mV/ms (time 2), 1.76 ± 0.95 mV/ms (time 3), *p* = 0.59. **(D)** Simultaneous optical and electrical AP recording in CA1 pyramidal cell. Top: Schematic illustration of patched cell and imaging region. Middle: Image of filled apical dendrite with highlighted region of interest (∑ = 130 pixels). Bottom: Simultaneous optical dendritic (red trace, DF/F = 3.2%, average of 2 trials) and electrical somatic (black trace, ΔV = 103.6 mV; Vm = −59.9 mV) recording. **(E)** Simultaneous optical and electrical EPSP/IPSP recording in CA1 pyramidal cell induced by SC stimulation. Top: Schematic illustration of patched cell, imaging region and stimulation site. Middle: Image of filled apical dendrite with highlighted region of interest (∑ = 121 pixels). Bottom: Simultaneous optical dendritic (red trace, DF/F (EPSP) = 0.6%, DF/F (IPSP) = 0.2%, average of 6 trials) and electrical somatic (black trace, ΔV (EPSP) = 12 mV, ΔV (IPSP) = 1.7 mV; Vm = −67 mV) recording. **(F)** DF/F vs. Vm correlation plot (taken from data of 10 cells) and the linear fit to this data (red). Note change in scaling on x- and y-axis. **(G)** High resolution optical recording after SC stimulation. Left: optical dendritic recordings of EPSP-IPSP patterns in the marked ROIs (1–5; average of 4 trials). Right top: Image of filled apical dendrite with highlighted ROIs. Right bottom: Optical recording over whole dendrite (∑ = 290 pixels). **(H)** Optical dendritic recordings of intracellularly induced action potential in the marked regions of interest (ROI) in same cell as in **(G)** (1–5; average of 10 trials). Bottom: Optical recording over whole dendrite (∑ = 290 pixels).

Since we were particularly interested in measuring the integration of synaptic signals in the dendritic tree with VSD imaging we tested the new red-shifted dye Di-2-ANBDQPTEA (Figures 2A,B) by loading hippocampal pyramidal cells via whole-cell patch clamp recording with intracellular solutions containing 0.10% dye (1 mg/ml) (Canepari et al., 2008, 2010). Cells were filled with the dye for an average of 40 min by whole-cell patch and kept for a further 40 min after the removal of the patch pipette for the whole dendritic tree to be sufficiently stained.

We measured action potential amplitudes, cell capacitance and input resistance at the beginning and the end of the loading phase; we did not detect any significant changes (Figure 2C left) in these parameters. Furthermore, the time courses of extracellularly evoked (inset Figure 2C) excitatory synaptic potentials did not change significantly while the cells were filling with the Di-2-ANBDQPTEA (Figure 2C right). After re-patching the cells, we either induced action potentials through somatic current injection or evoked EPSP-IPSP sequences by extracellular stimulation of Schaffer collaterals (Figures 2D,E top). We measured the fluorescence changes over the whole visible dendritic tree (Figures 2D,E middle). Comparison of somatic whole-cell recordings and averaged dendritic VSD imaging reveals tightly correlated responses to either somatic action potential induction or synaptic signals (Figures 2D,E bottom). Synaptic signals could be blocked by a combination of NBQX (20 µM) and APV (100 µM) for depolarizing and bicuculline (20 µM) for hyperpolarizing responses; indicating that these were ionotropic glutamatergic, respectively GABAergic postsynaptic potentials (Figures 4A,B). When comparing the magnitude of fluorescence changes in % DF/F with the membrane potential change recorded with the somatic electrode for all these signals, we observed a roughly linear relationship (Figure 2F; *r* = 0.86, *N* = 11) with a slope of 0.33 DF/F per Volt membrane potential change. To assess the capability of the dye to resolve transmembrane voltage transients at a subcellular level, we performed the same experiments (as in Figures 2D,E), but segmented the dendritic tree into different ROIs. As can be seen (Figure 2G) EPSP-IPSP sequences could be reliably imaged in ROIs consisting of 20–60 pixels. Backpropagation of somatically evoked action potentials could be imaged in the same ROIs (Figure 2H). Thus VSD imaging with Di-2-ANBDQPTEA can resolve subthreshold and suprathreshold membrane voltage transients at a subcellular level.

**Figure 3:**
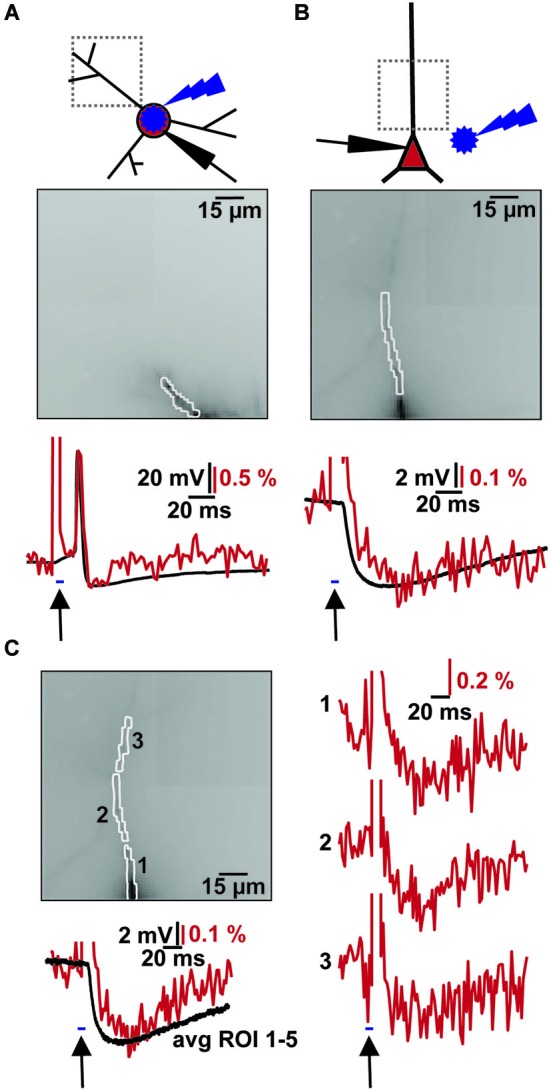
**Combined VSD recording with ChR2 activation. (A)** Imaging of light evoked AP in ChR2+ interneuron. Top: Schematic illustration of patched ChR2+ interneuron, site of blue flash and imaging region. Middle: Image of filled dendrite with highlighted region of interest (∑ = 54 pixel). Bottom: Simultaneous optical dendritic (red trace, DF/F = 2.1%, average of 2 trials) and electrical somatic (black trace, ΔV = 73.4 mV; Vm = −40 mV) recording. Arrow indicates blue light application (2 ms). **(B)** Imaging of induced IPSP in CA1 pyramidal cell. Top: Schematic illustration of patched cell, site of blue flash and imaging region. Middle: Image of filled apical dendrite with highlighted region of interest (∑ = 34 pixel). Bottom: Simultaneous optical dendritic (red trace, DF/F (IPSP) = 0.4%, average of 10 trials) and electrical somatic (black trace, ΔV (IPSP) = 4.9 mV; Vm = −60.1 mV) recording. Arrow indicates blue light application (5 ms). **(C)** High resolution optical recording after blue light stimulation. Left top: Image of filled apical dendrite with highlighted ROIs (1–3, average of 6 trials). Left bottom: Simultaneous optical dendritic (red trace, ∑ = 130 pixels, DF/F (IPSP) = 0.3%) and electrical somatic (black trace, ΔV (IPSP) = 5.9 mV; Vm = −59.8 mV) recording. Right: Optical dendritic recordings of evoked IPSP patterns in the marked ROIs. Arrow indicates blue light application (5 ms).

**Figure 4:**
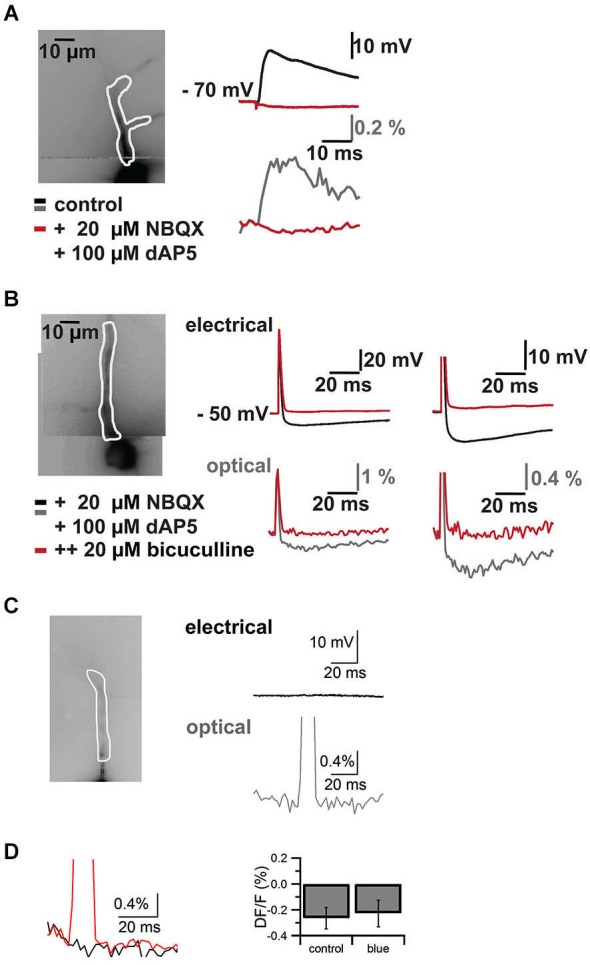
**Control experiments**. Pharmacological block of synaptic signals in cells filled with VSD and blue laser artifact in the absence of an electrical response. Responses are recorded both with somatic patch clamp electrodes (top traces in mV) and by VSD imaging (bottom traces in% DF/F). **(A)** Imaged neuron with ROI on apical dendrite shown in white outline on the left. On the right EPSP through the somatic patch clamp electrode top and VSD imaging in the region of interest bottom. Black/gray traces are baseline, red traces are after addition of the NMDA and AMPA receptors AP5 and NBQX respectively. **(B)** Imaged neuron with region of interest on apical dendrite in white outline on the left. A stimulus electrode was placed near the soma of the neuron to evoke an action potential and an inhibitory postsynaptic potential (excitatory synaptic transmission was blocked with AP5 and NBQX). Traces on the right show electrode recordings on top and VSD imaging at bottom at lower (left) and higher resolution (right). Black/gray are baseline traces, red traces are after application of the GABA(A) receptor antagonist bicuculline. Note that the action potential is preserved and faithfully recorded by VSD, while the inhibitory synaptic signal is completely blocked by the GABA(A) receptor antagonist. I four such cells we could completely block synaptic signals with a combination of AP5, NBQX and bicuculline. **(C)** Imaged neuron with region of interest overlaid on the left. Electrical recording (top, black) and optical recording (bottom, gray) from a CA1 pyramidal neuron. The focused laser beam failed to hit ChR2 expressing structures and thus failed to elicit a response. A square pulse artifact can be observed in the optical trace. **(D)** Left, overlay of trace without laser stimulation (black) and with laser stimulation (red) in the absence of a synaptic response. The decay in the response is due to dye bleaching, which was not compensated in this case. To quantify the effect of the blue laser pulse in the absence of synaptic responses, we measured the difference between two 5 ms epochs, one 5 ms before the pulse and one directly following the laser pulse. We compared control trials (DF/F −0.27 ± 0.08%) without laser pulse and trials with laser pulses (DF/F −0.23 ± 0.13%) (*N* = 4, *p* > 0.8).

Combining the two techniques allowed us to measure the effects of ChR2 activation on GABAergic cells and to study synaptic responses in target cells (Figure 3). We filled interneurons with VSD and illuminated them with brief blue laser flashes during VSD imaging (Figure 3A top). Action potentials could be imaged in the dendritic tree (Figure 3A middle) and compared to somatic whole-cell recordings (el: 78.2 ± 6.7 mV, opt: 1.6 ± 0.7 DF/F; *N* = 2; Figure 3A bottom). Brief 473 nm illumination of the slice in the perisomatic region of dye-filled pyramidal cells evoked IPSPs that could be detected both, by VSD imaging and somatic whole-cell recordings (el: 5.4 ± 1.2 mV, opt: 0.4 ± 0.1 DF/F; *N* = 3; Figure 3B bottom). Again, the signals could be resolved at a subcellular level (Figure 3C). Due to blue light absorption by the VSD, the resulting fluorescence change was visible in the optical recording and the resulting short artifact precluded correct interpretation of VSD fluorescence during blue laser application. The artifact did not outlast the blue laser application, as seen in experiments in which the blue laser illumination failed to generate a synaptic response (Figures 4C,D).

## Discussion

Here we demonstrate the function of an optical system to combine optogenetic activation of defined neurons and high-resolution single cell VSD imaging.

Previously, we have achieved combinations of VSD- and calcium imaging and caged-compound photolysis using near-UV excitation for the secondary imaging component (Vogt et al., 2011a,b). Excitation of ChR2 cannot be performed in combination with imaging cells with the dye (JPW1114) used in these experiments, due to significant activation of ChR2 with the excitation illumination of JPW1114. Recent optical approaches have successfully combined extracellular loading of red-shifted VSDs with ChR2 stimulation to study signal propagation in networks (Lim et al., 2012; Tsuda et al., 2013). The technique presented here is technically more demanding than bulk loading, since patch-clamp recordings have to be obtained and the cells ideally have to be re-sealed by obtaining the outside-out configuration through careful removal of the pipette.

Here, we have now achieved a combined VSD-imaging and ChR2(H134R) excitation after cell loading through patch pipettes by using a newly developed VSD, Di-2-ANBDQPTEA (Yan et al., 2012). We demonstrate that this allows measuring of IPSPs at a subcellular resolution after ChR2 activation. The signal to noise ratio in these experiments also allows resolving of inhibitory and excitatory postsynaptic potentials in subcellular compartments after extracellular stimulation as demonstrated before with other VSD dyes (Canepari et al., 2010). Our experiments were performed at room temperature. While the dye has been used at elevated temperatures before (Leão et al., 2012) and related dyes at near physiological temperatures (Canepari et al., 2008), the performance of this dye at physiological temperatures needs to be established. Di-2-ANBDQPTEA absorbs light at 473 nm and this results in optical signals if stained cells are illuminated by blue light. The impact of these artifacts can be minimized by spatially separating ChR2 stimulation from VSD imaging and by using short blue light pulses. Even a direct blue light stimulation of a VSD loaded interneuron disturbed the fluorescence signal only for the duration of the light pulse, allowing the detection of the resulting slightly delayed action potential through VSD fluorescence.

Prolonged illumination of Di-2-ANBDQPTEA as well as many other VSDs at their excitation wavelength will cause considerable bleaching and potentially phototoxicity. Care should be taken to limit the exposure to a minimum. This technique is therefore not suitable for the measurement of very slow processes or of spontaneous signals.

Increasing evidence show that the function of neurons in a network is linked to their molecular and developmental identity (Ko et al., 2013). This is particularly true for inhibitory interneurons, which have long been classified according to a series of molecular markers (DeFelipe et al., 2013). Selective ChR2 expression makes it possible to activate a particular subclass of interneurons in precisely defined temporal patterns (Atallah et al., 2012). Different subtypes of interneurons are also characterized by their precise subcellular target area (Group PIC, 2008). The functional consequences of this selective innervation are not fully understood. VSD-imaging with subcellular resolution will allow a detailed analysis of the impact of signals from defined interneurons on dendritic signal-propagation and -integration. In addition, as shown previously (Canepari et al., 2010) this can be achieved without distorting the physiological ion gradients in the target neurons.

In our experiments, we have often been able to evoke inhibitory responses in target neurons also just by focal illumination of the neuropil close to the imaged cells. This is likely due to the high density of ChR2 expressing axons surrounding the target cells. This complicates the identification of the origin of the signals and thereby the identification of interneuron subtypes. A more sparse expression of ChR2 in just a subpopulation of interneurons would improve this considerably. While the diameter of the illuminated spot in the focal plane was around 30 µm, significant scattering of the blue laser light in the slice tissue was observed. Again a sparse ChR2 expression will help avoiding spurious activation of neurons due to scattered light.

As with JPW1114, resolving synaptic signals requires high intensity illumination of the preparation with a stable light source. As before, solid-state lasers proved to be ideal for the task (Canepari et al., 2010). Photo damage is a significant concern in such experiments. Care should be taken to monitor the health of the cells and the stability of the studied signals to avoid artifacts due to dye degradation.

Our experiments demonstrate that a study of neural networks based solely on optical recording is possible. This holds the promise of a rapid analysis of a network’s specific connectivity and function, including dendritic processing in its neurons.

## Author contributions

Experiments were designed by Silvia Willadt and Kaspar E. Vogt. Data was collected and analyzed by Silvia Willadt and Kaspar E. Vogt. The article was written and revised by Silvia Willadt, Marco Canepari, Ping Yan, Leslie M. Loew and Kaspar E. Vogt. All authors read and approved the final version of the manuscript.

## Conflict of interest statement

The authors declare that the research was conducted in the absence of any commercial or financial relationships that could be construed as a potential conflict of interest.
